# Digital Transformation of Healthcare Access: A Comparative Time Series Analysis of Online Versus Conventional OPD Registrations at a Tertiary Care Hospital

**DOI:** 10.7759/cureus.81970

**Published:** 2025-04-09

**Authors:** Ayushi Ranjan, Geetu Singh, Himalaya Singh, Mohit Singh

**Affiliations:** 1 Community Medicine, Sarojini Naidu Medical College, Agra, IND; 2 Community Medicine, Ayushman Bharat Digital Mission (ABDM), Lucknow, IND

**Keywords:** ayushman bharat digital mission, digital health registration, digital transformation, healthcare process efficiency, time motion study

## Abstract

Introduction: The Ayushman Bharat Digital Mission (ABDM) is transforming India's healthcare through digitization, including online outpatient department (OPD) registration using Ayushman Bharat Health Account (ABHA) identities (IDs). With an increase in the number of ABHA accounts, understanding time requirements and bottlenecks in these digital workflows is crucial for successful implementation.

Primary objective: To evaluate the efficiency of patient registration by measuring and comparing the time taken for each step in the online registration and conventional offline OPD registration.

Secondary objective: To determine ARIMA (autoregressive integrated moving average) forecasting models to predict future registration and waiting times based on historical data patterns.

Methods: This cross-sectional study observed 40 patients (20 per registration method) using Time & Motion Study software. Data collection included four process steps: arrival, application download/token generation, waiting, and registration. Analysis used descriptive statistics, the Mann-Whitney U test, and ARIMA modeling.

Results: Online ABHA registration averaged 4.15 minutes (SD = 1.28) versus 10.37 minutes (SD = 3.54) for offline registration, representing approximately 60% time savings. This difference was statistically significant (W = 16, p < 0.01). The main bottleneck was waiting time: nine minutes for offline versus one minute for online registration. ARIMA models predicted continued efficiency with a four-minute average time for online registration compared to 12 minutes for offline, with greater variability in the latter.

Conclusion: ABHA online registration demonstrates significantly improved efficiency and consistency over conventional methods, particularly in reducing waiting times. These findings support continued implementation of digital registration systems while highlighting the need for strategies to manage process variability in both systems.

## Introduction

Digital transformation in healthcare refers to technological innovations that improve service delivery and benefit society. This transformation incorporates internet-based solutions, emerging technologies, and evidence-based practices to improve health management procedures [1]. The spectrum of digital health includes telemedicine, e-health, m-health, and health informatics systems working in concert to address medical challenges. In pursuit of Sustainable Development Goals (SDGs), particularly SDG 3, United Nations agencies, member states, and various organizations are strategically integrating technology into healthcare infrastructure to achieve universal health coverage. Digital health strengthens health systems while enhancing health promotion and disease prevention strategies, making it instrumental in realizing the SDGs [2,3]. Gjellebæk et al. argue that new digital technologies will shift healthcare toward digitalization, bringing significant benefits to patients and healthcare infrastructure [4]. The healthcare landscape in India is undergoing a significant digital transformation through the Ayushman Bharat Digital Mission (ABDM). This nationwide initiative aims to digitize healthcare services and create a unified health ecosystem that seamlessly connects patients, healthcare providers, and medical facilities. At the forefront of this transformation is the implementation of online outpatient department (OPD) registration using Ayushman Bharat Health Account (ABHA) identities (IDs) in place of conventional offline OPD registration processes, which are fundamentally changing how patients interact with healthcare facilities [5]. As of September 30, 2024, more than 67 crore ABHA IDs have been created in India [6]. The transition from traditional paper-based systems to digital registration processes represents both an opportunity and a challenge for healthcare facilities. While digital systems promise improved efficiency, reduced errors, and better data management, their implementation requires careful consideration of operational aspects, user experience, and resource allocation [7]. Understanding time requirements and identifying bottlenecks in digital workflows is crucial for successful adoption and patient satisfaction.

Time-motion studies (TMSs) are essential to improve working efficiency in healthcare settings, providing quantitative metrics for process optimization [5]. By conducting detailed observations of these processes, the study aims to provide valuable insights into the operational efficiency of digital registration workflows. There are also tools for future predictions, one of which is ARIMA (autoregressive integrated moving average) [8]. It helps forecast trends in registration workflows by analyzing time-series data to predict patient registration volumes, identify peak periods, and detect bottlenecks. The scope of this study encompasses several key aspects, such as the time required for various steps in online ABHA ID creation or token generation, duration of the offline OPD registration process, and the impact of digital processes on overall patient flow.

Understanding these temporal aspects is crucial for several reasons. First, it helps identify areas where the registration process can be streamlined or improved [9]. Second, it provides concrete data for resource allocation and staff training needs [10]. Third, it offers insights into the real-world implementation challenges of digital health initiatives, which can inform future improvements and policy decisions. The findings from this study will be particularly valuable for healthcare administrators and policymakers involved in ABDM implementation. By quantifying the time requirements and identifying operational challenges, this research will contribute to developing more efficient registration processes, reducing patient waiting times, and improving the overall quality of healthcare service delivery [9].

This TMS represents an important step in understanding and optimizing the digital transformation of healthcare registration processes. The insights gained will not only benefit individual healthcare facilities but also contribute to the broader success of India's digital health initiative. India's ABDM is a major step toward creating a complete digital healthcare system for the country [7]. At the core of this mission lies the creation of a unique online ABHA and the digital registration of patients in OPDs. While this digital transformation promises enhanced healthcare delivery and seamless patient experiences, understanding its operational efficiency is crucial for successful implementation.

Primary objective

To evaluate the efficiency of patient registration by measuring and comparing the time taken for each step in the online registration and conventional offline OPD registration.

Secondary objective

To determine ARIMA forecasting models to predict future registration and waiting times based on historical data patterns.

## Materials and methods

Study design and setting

This study employs a cross-sectional observational design utilizing time-motion analysis between the ABHA OPD registration and the conventional offline OPD process. The study was conducted in the OPD registration complex of a hospital, where both online and conventional offline OPD registrations are performed. Before data collection, permission was obtained from the OPD administration. Since the research involved only passive observation of operational workflows without collecting patient-identifiable information or requiring direct interaction with patients, separate institutional ethical clearance was not required. Data collection was conducted through systematic observation using Time & Motion Study software version 2.0.1 for precise time measurements [11]. A standardized data collection template was developed to capture the duration of each process step. The template includes dedicated cyclical elements for recording start times, end times, and total duration in seconds for each component of the registration process (Appendices).

Sampling strategy

The study population comprised patients visiting the OPD. A total of 40 patients were included, based on operational feasibility and ensuring sufficient representation of the diverse OPD population. While previous time-motion studies in healthcare settings with similar objectives have used a sample size of 30 patients, we opted for a slightly larger number to account for variations in patient flow and system performance and to enhance representativeness [12]. Data collection was conducted from October 1st to October 21st, 2024. To minimize selection bias and ensure fair representation across time, four patients were randomly selected (two from each registration process) between 9 am and 12 pm. At each hourly interval (9 am, 10 am, 11 am, and 12 pm), one patient was selected using simple random sampling. Patients coming for follow-up or revisits were excluded. This approach allowed for a random yet representative selection of subjects across different times of operation.

Outcome measures

**Figure 1:**
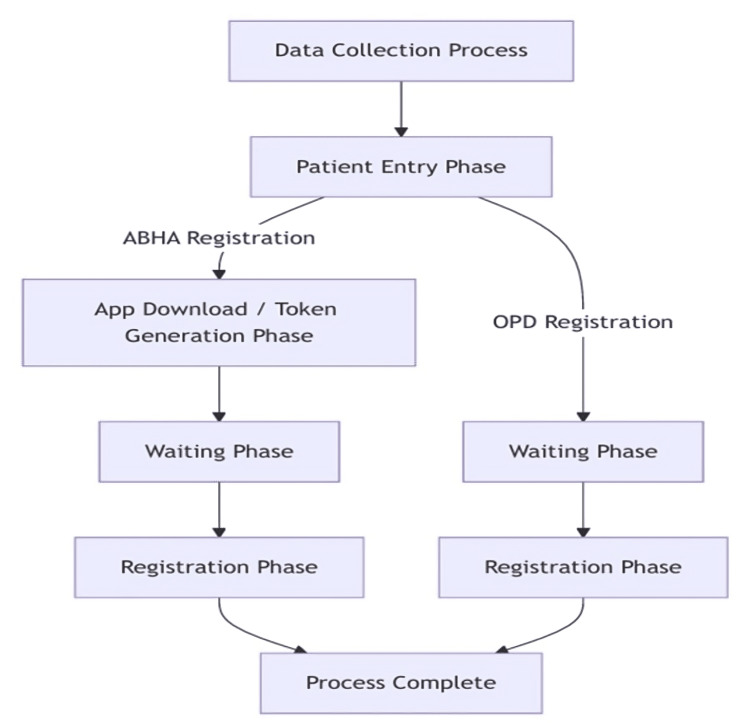
Comparative workflow of online ABHA and conventional offline OPD registration processes. ABHA: Ayushman Bharat Health Account; OPD: outpatient department.

The key outcome measures focused on the duration of each process step in both the online ABHA registration and offline OPD processes. The key steps being monitored include patient arrival, government mobile applications download or token number generation, waiting time, and registration, as shown in Figure 1. Inter-observer reliability was established through a pilot observation of five patients prior to the main study, achieving a concordance rate of >95% in timing measurements. For each observation, the observer was positioned strategically in the registration area to maintain a clear view of all process steps while avoiding interference with normal operations. Real-time measurements of each step were recorded using the time-motion software, with immediate entry into the data collection template. This methodical approach to timing each component of the registration process enabled detailed analysis of workflow efficiency and identification of potential bottlenecks in the system.

Confounding factors

Several factors may have influenced the registration times observed in this study. Variations in internet connectivity could have impacted the efficiency of the online ABHA registration process, as no measures were taken to standardize network conditions, potentially introducing variability. Patient digital literacy also played a role, with individuals familiar with digital health applications likely completing the process faster than those less experienced. Additionally, time-of-day variations may have influenced registration times due to fluctuations in patient volume and workload across different time slots. While these factors were not controlled, acknowledging them provides context for interpreting our findings. Future studies could incorporate strategies to minimize these confounders for a more standardized comparison.

Data quality assurance

To ensure data quality and consistency, the observer underwent standardized training using recordings of registration processes. Periodic cross-verification of timing data was performed between observers.

Statistical analysis

The analysis involved several statistical tests and was done using R version 4.4.1 (R Foundation for Statistical Computing, Vienna, Austria) [13]. First, descriptive statistics were calculated, including the mean, median, standard deviation (SD), and standard error (SE), to summarize the data for online ABHA and offline OPD registration times. The Shapiro-Wilk test for normality was then conducted to check if the data followed a normal distribution, which was confirmed and indicated that the data are not normally distributed. After which Mann-Whitney U test was applied after determining that the data (online ABHA total time and offline OPD total time) did not follow a normal distribution, with the level of significance demarcated at P < 0.05. The time series data for both online ABHA and offline OPD registration processes exhibited stationarity as confirmed by the augmented Dickey-Fuller test, enabling the subsequent application of ARIMA modeling. ARIMA forecasting models were applied to predict future registration times for both systems, providing insights into their stability and variability [8]. Finally, the autocorrelation function (ACF) was used to examine the residuals of the forecasting models, ensuring that no significant autocorrelations remained, indicating that the models effectively captured the data's underlying patterns [8].

## Results

The descriptive statistics in Table 1 for the two groups show that the online ABHA ID registration process times have a mean of 4.15 minutes with a median of 3.77 minutes, indicating a relatively consistent and lower registration time compared to offline OPD registration process, which has a mean of 10.37 minutes and a median of 10.89 minutes. The standard deviation for online ABHA is 1.28 minutes, suggesting less variability in registration times, while the offline OPD group has a higher standard deviation of 3.54 minutes, indicating greater variability in the registration process. The standard error for online ABHA is smaller (0.287 minutes), implying a more precise estimate of the mean compared to offline OPD's standard error of 0.791 minutes. Overall, these statistics suggest that online ABHA registration times are not only faster but also more consistent than offline OPD registration times.

**Table 1:** Descriptive statistics of registration times for online ABHA and conventional offline OPD. All time measurements are in minutes. Data collected through the time-motion study of patient registration processes. n: sample size; SD: standard deviation; SE: standard error; ABHA: Ayushman Bharat Health Account; OPD: outpatient department.

Descriptive statistics					
Type of OPD registration	n	Mean	Median	SD	SE
Online ABHA ID registration	20	4.15	3.77	1.28	0.287
Offline OPD registration	20	10.37	10.89	3.54	0.791

**Figure 2:**
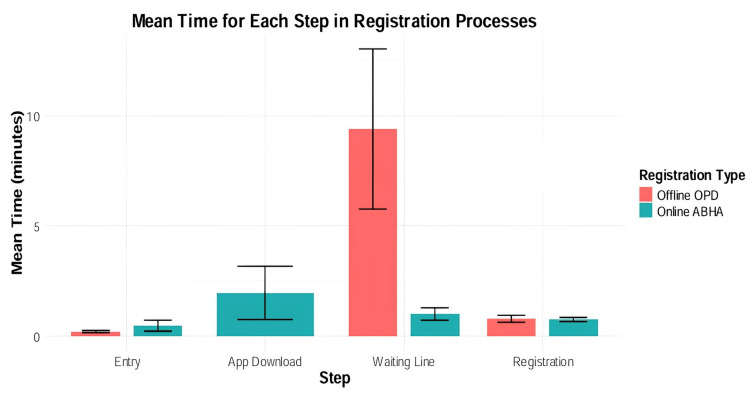
Mean time distribution across registration cycle phases for online ABHA and conventional offline OPD. ABHA: Ayushman Bharat Health Account; OPD: outpatient department.

The graph in Figure 2 breaks down the average time taken for each step in both registration processes. The entry step takes approximately 0.2 minutes for both offline OPD and online ABHA. The app download and data entry step, unique to online ABHA, requires about two minutes. The difference appears in the waiting line time, where offline OPD patients spend roughly nine minutes while online ABHA users wait only about one minute. The final registration step is similar for both systems, taking approximately 0.8 minutes each.

**Figure 3:**
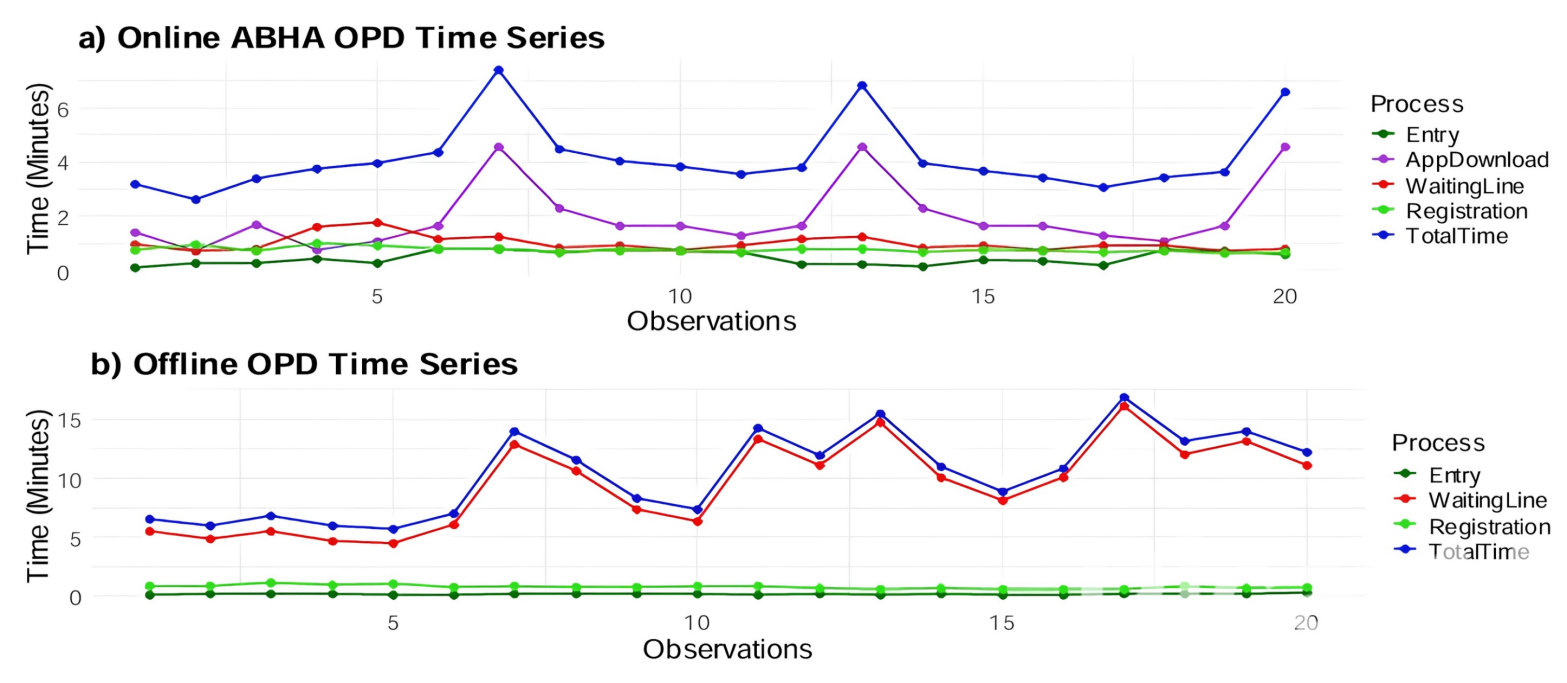
Time series plot of total registration times. (a) ABHA ID registration time series shows the time required for different stages in the ABHA registration process, including app download (purple), registration (green), total time (blue), and waiting line (red). (b) General OPD time series displays OPD visit times, including registration (green), total time (blue), and waiting line (red). ABHA: Ayushman Bharat Health Account; OPD: outpatient department.

The comparative time series analysis between traditional offline OPD and digital ABHA ID registration systems reveals significant contrasts in operational efficiency patterns, as illustrated in Figure 3. In the offline OPD system, the total time and waiting time show a strong correlation, with peaks reaching 15-17 minutes, while registration time remains consistently low at one to two minutes. This suggests that waiting time is the primary bottleneck in the OPD process. The online ABHA registration process, on the other hand, demonstrates a different pattern with three distinct spikes in total time reaching up to eight minutes. While its registration and app download/token generation times remain relatively stable, the waiting time appears to be the main contributor to these peaks. Both processes indicate that the core registration activities are well-optimized, but the offline OPD system faces challenges with waiting times, possibly due to resource constraints or peak load issues. The online ABHA process generally shows lower overall processing times despite occasional spikes, whereas the offline OPD process demonstrates more consistent but higher total times throughout the observations.

**Figure 4:**
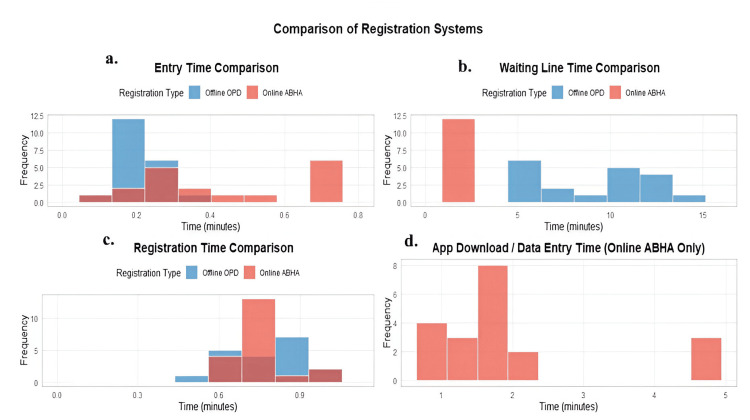
Histogram distribution analysis of registration process components: online ABHA and conventional offline OPD. Four-panel histogram set showing frequency distributions of registration times. Panels include: (a) entry time comparing initial processing durations; (b) waiting line time showing queue duration patterns; (c) registration time comparing final processing efficiency; and (d) app download/data entry time specific to ABHA implementation. Time is measured in minutes. Frequency indicates occurrence count. Both systems are represented with distinctive colors: offline OPD (light blue) and online ABHA (coral), with overlapping distributions shown transparently. ABHA: Ayushman Bharat Health Account; OPD: outpatient department.

In Figure 4, the detailed time distributions reveal interesting patterns across four key metrics. The entry time distribution shows offline OPD clustered around 0.2 minutes, while online ABHA spreads between 0.2 and 0.8 minutes. The waiting line distribution is particularly telling offline OPD times scatter between five and 15 minutes, while online ABHA consistently shows shorter times around two to three minutes. Registration time distributions for both systems cluster between 0.6 and 0.9 minutes. The app download/data entry step, exclusive to online ABHA, typically takes one to five minutes, with most cases concentrated around two minutes.

**Figure 5:**
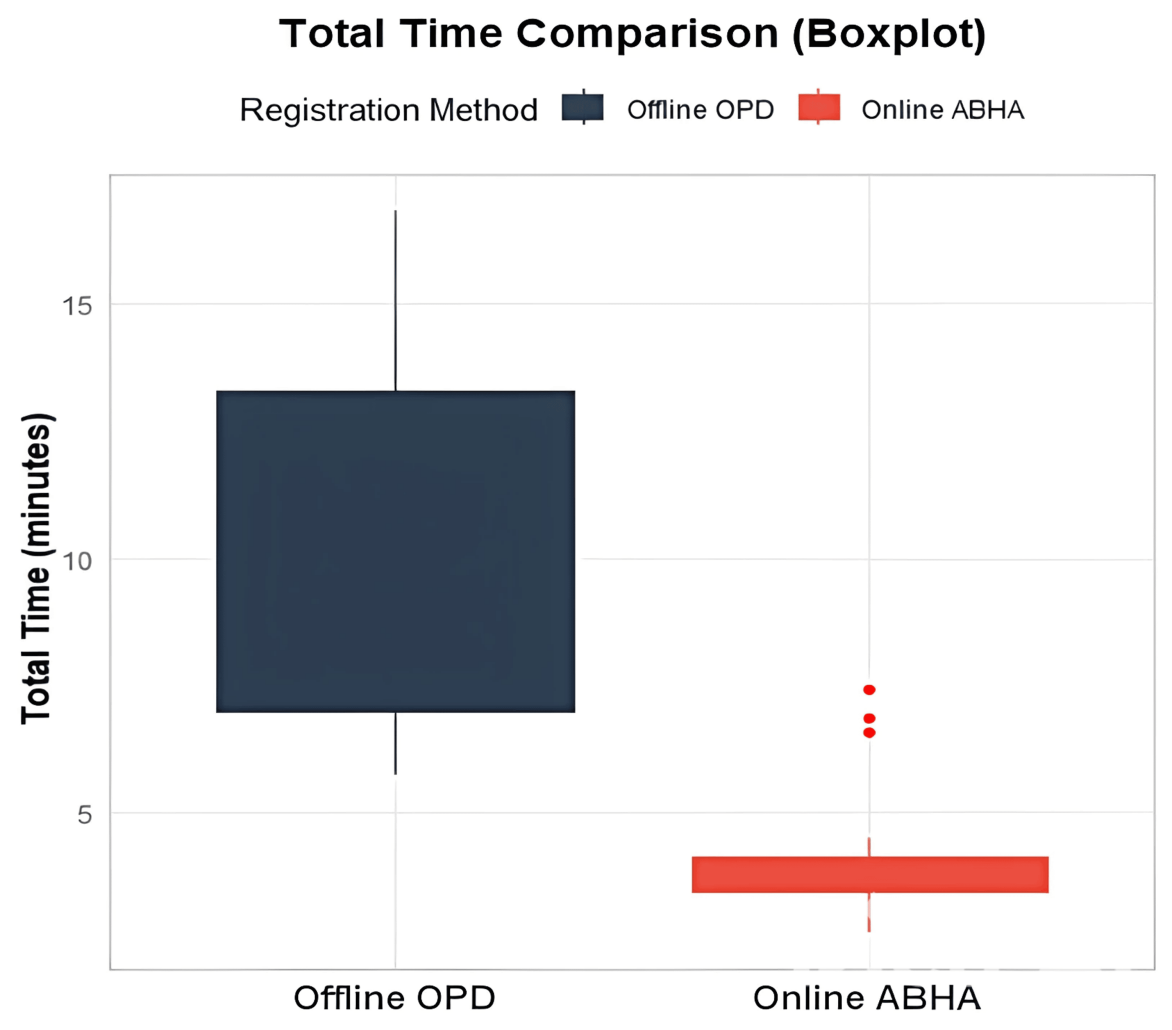
Boxplot comparison of total time of online ABHA and conventional offline OPD. ABHA: Ayushman Bharat Health Account; OPD: outpatient department.

In Figure 5, the comprehensive time comparison shows a stark contrast between the two systems. Offline OPD demonstrates a median total time of approximately 10-11 minutes, with a broader range extending from seven to 13 minutes, indicating higher variability. In contrast, online ABHA maintains a median time of about four minutes, with a more compressed range, suggesting greater consistency. The online system does show a few outlier cases reaching up to seven to eight minutes, but these are exceptional rather than typical. The boxplot clearly illustrates that online ABHA achieves time savings of roughly six to seven minutes compared to the traditional offline OPD system.

The results in Table 2 of the Mann-Whitney U test indicate a statistically significant difference in the registration times between the online ABHA and offline OPD groups. The test statistic (W = 16) reflects a measure of the difference between the groups. With a p-value < 0.01, which is smaller than p < 0.05, the test strongly rejects the null hypothesis, suggesting that the observed difference is highly unlikely to have occurred by chance. The alternative hypothesis, stating that the two groups have different distributions of registration times, is supported. This finding points to a significant disparity in the registration processes of online ABHA and offline OPD, implying that the efficiency or organization of these processes differs. The results highlight the need to explore potential areas for improvement, whether in streamlining the online ABHA registration system or optimizing the OPD process, to enhance overall efficiency and reduce waiting times.

**Table 2:** Mann-Whitney U test. * Data represents the total time taken for registration processes under online ABHA and offline OPD categories, measured in minutes. The p-value indicates a statistically significant p < 0.05. ABHA: Ayushman Bharat Health Account; OPD: outpatient department.

Test	Variable	W statistic	p-value	Alternative hypothesis
Mann-Whitney U test	Online ABHA total time and offline OPD total time	16	<0.01	True location shift is not equal to 0

**Figure 6:**
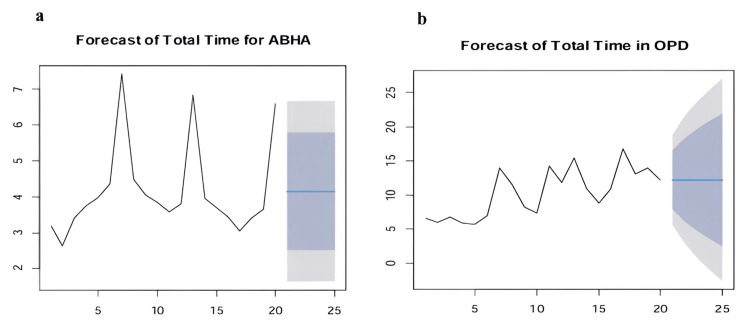
ARIMA forecast of total registration time. (a) ABHA ARIMA forecast. (b) OPD ARIMA forecast. Time series forecast showing historical offline OPD and online ABHA registration times (black line) and predicted values (blue line) with 80% (darker shade) and 95% (lighter shade) confidence intervals. Time points on the x-axis and registration time in minutes on the y-axis. ARIMA: autoregressive integrated moving average; ABHA: Ayushman Bharat Health Account; OPD: outpatient department.

The forecast graphs in Figure 6 for total time in offline OPD and online ABHA reveal distinct patterns and implications. The offline OPD graph demonstrates historical fluctuations between approximately five to 17 minutes, with an overall increasing trend toward the 20th data point. The forecast suggests a central prediction of around 12 minutes but with significant uncertainty, as evidenced by widening confidence intervals, indicating possible wait times up to 25 minutes in the worst-case scenario. In contrast, the online ABHA graph exhibits a more volatile pattern with sharp peaks, notably around the 7th and 15th data points, reaching about seven minutes. The forecast projects a central prediction of approximately four minutes, with a narrower range of potential outcomes between two and six minutes compared to offline OPD. While online ABHA times appear more manageable due to the constrained forecast range, offline OPD times are a greater concern, showing higher absolute values and variability. The widening confidence intervals in both forecasts highlight the growing uncertainty over time, emphasizing the need for strategies to mitigate underlying process variability and improve predictability.

**Figure 7:**
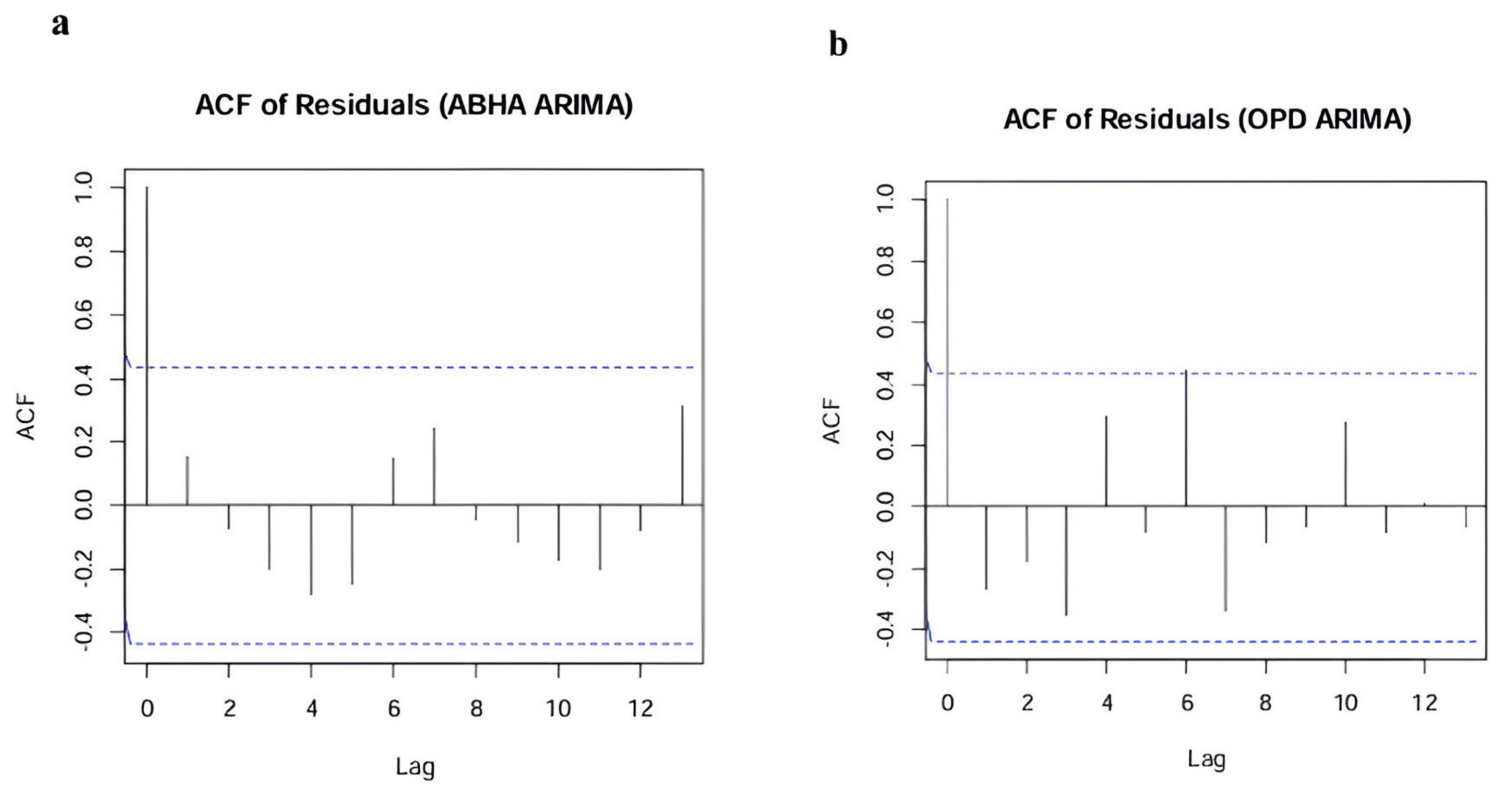
Autocorrelation function (ACF) analysis of ARIMA model residuals. (a) ACF residual online ABHA. (b) ACF residual offline OPD. ACF plot showing correlation of residuals at different time lags. Dashed blue lines represent significance bounds (±0.4). Spikes extending beyond these bounds indicate potentially significant autocorrelations. Values between -1 and 1 on the y-axis represent correlation strength. ARIMA: autoregressive integrated moving average; ABHA: Ayushman Bharat Health Account; OPD: outpatient department.

The ACF of residuals in Figure 7 for both offline OPD and online ABHA ARIMA models shows key insights into model performance and areas for potential improvement. Both graphs feature dashed significance bounds at approximately ±0.4, with lag values ranging from 0 to 13 and an initial spike at lag 0, which is expected and equal to 1.0. For the offline OPD ARIMA residuals, most autocorrelations fall within the significance bounds, but there are notable spikes at lag 6 (positive correlation), lag 1 (negative correlation), and lag 10 (positive correlation). These spikes suggest some periodic structure that the model has not fully captured. In contrast, the online ABHA ARIMA residuals show a generally more random pattern, with stronger negative correlations at lags 4-5 and positive spikes at lags 8 and 13. This random pattern indicates that the online ABHA model has more effectively captured the underlying data structure. Overall, while both models perform adequately with most correlations within bounds, the offline OPD model shows residual periodicity that could benefit from further refinement, whereas the online ABHA model appears to be more accurate in modeling the data.

## Discussion

The present time-motion study investigating the ABDM digital transformation initiative shares methodological foundations with several healthcare efficiency studies conducted across India, while introducing unique elements specific to digital healthcare transformation. Our methodology aligned with approaches used by Alekhya et al. (2022) and Khot et al. (2023), employing direct observation techniques to record precise timing data [9,14]. However, our study's distinctive focus on comparing parallel registration systems (online ABHA and offline OPD) provided insights that were not captured in previous research. While researchers like Karthikeyan et al. (2024) and Sengupta et al. (2020) examined broader patient flows through offline OPD services, our targeted investigation of the registration process enabled a detailed analysis of this crucial first point of contact [15,16].

The results of our study demonstrate significant improvements in processing efficiency through digital transformation. The offline OPD registration time of 10.37 minutes aligns with findings from previous studies, such as Khot et al. (2023), who reported 94% of registrations completed within 10 minutes, and Yadav (2024), who found an average wait time of 8.31 minutes [9,17]. However, our online ABHA system's average processing time of 4.15 minutes represents a marked improvement over both our traditional system and those reported in other studies, even while accounting for the additional step of app download and token generation (1.95 minutes).

The reduction in waiting times achieved with the online ABHA system (0.99 minutes versus 9.39 minutes) represents a more substantial improvement than those achieved through traditional optimization methods. This becomes particularly evident when compared to studies like Naaz et al. (2019), who reported total OPD times of approximately two hours, and Sengupta et al. (2020), who found patients spending an average of one hour and 50 minutes in offline OPD waiting lines [16,18]. Furthermore, the greater consistency in online ABHA processing times (SD = 1.28 versus 3.54 for OPD) suggests that digital transformation offers more standardized service delivery than traditional approaches.

The implementation of digital transformation in healthcare registration processes presents both opportunities and challenges that differ from traditional optimization approaches. While previous studies, such as Ahmad et al. (2017), focused on staff reallocation and queue management, our findings highlight the need for comprehensive technical infrastructure and support systems [10]. The success of digital transformation requires careful attention to patient education and support, particularly regarding the initial app download and registration process, an aspect not addressed in previous studies focusing solely on workflow optimization.

Based on our comparative analysis, healthcare facilities considering digital transformation should adopt a phased approach, initially maintaining both traditional and digital systems to ensure service continuity. This should be accompanied by robust technical support systems and systematic monitoring of both processing and waiting times. Resource optimization, particularly during peak hours, remains crucial but requires new considerations in the digital context.

Notably, our analysis suggests that online ABHA registration times could be even faster with optimal internet connectivity. During periods of stable, high-speed internet access, registration times showed potential to decrease further, potentially reaching sub-four-minute processing times. This observation indicates that the current average processing time of 4.15 minutes may actually represent a conservative estimate of the system's potential efficiency, with network stability being a key factor in maximizing the benefits of digital transformation.

Limitations

The sample size of 40 patients, while sufficient for statistical significance, represents a limited temporal snapshot. A larger sample collected over an extended period would better account for seasonal variations in patient flow and system performance. Additionally, the novelty of the ABHA system means that our data represents early-stage implementation results, and long-term efficiency patterns may differ as both staff and patients become more familiar with the system. The study also did not account for potential geographical variations in patient flow, which could affect processing times differently in digital versus traditional systems.

Recommendations

Healthcare facilities implementing digital transformation should invest in robust internet infrastructure, including redundant high-speed fiber connections from providers like internet fiber, along with 4G/5G cellular failover systems. Healthcare facilities should adopt a phased hybrid implementation approach initially, maintaining both traditional and digital systems with integration tools compatible with India's National Informatics Centre (NIC)-developed systems and the ABDM ecosystem. They should establish tiered technical support with on-site IT specialists utilizing affordable remote support tools like AnyDesk or TeamViewer, Information Technology Infrastructure Library (ITIL)-based help desk services from India, and comprehensive staff training programs through platforms or government-sponsored digital literacy initiatives.

Unlike traditional optimization focused solely on workflow, digital transformation requires both technical expertise and enhanced service capabilities. Real-time monitoring dashboards should be implemented using affordable solutions like network monitors or open-source alternatives like Zabbix to track processing times, waiting periods, and technical performance, particularly during peak hours. Automated alerts should be configured for internet performance metrics through WhatsApp Business API (application programming interface) or SMS (short message service) gateways to immediately identify potential bottlenecks in the NIC network infrastructure.

Tailored digital literacy initiatives should be developed, including multilingual tutorials in Hindi, English, and regional languages, pictorial step-by-step printed guides, and dedicated assistance for first-time users. Guided tutorials should be implemented within the ABHA application itself and leverage India's robust common service centers (CSCs) network to provide hands-on support in rural areas. These targeted interventions should enable healthcare facilities to successfully implement digital transformation while maintaining service quality and improving operational efficiency within India's unique healthcare ecosystem.

## Conclusions

This comprehensive time-motion observational study reveals the profound impact of digital transformation on healthcare accessibility, demonstrating how technological innovation fundamentally restructures the patient registration experience. The comparative observation demonstrates that technological innovation in registration processes can substantially reduce administrative burdens for both patients and healthcare providers alike. The consistency observed in the digital pathway suggests a more standardized and predictable experience for healthcare consumers. The transition toward digitized healthcare administration represents a significant paradigm shift in how patients interact with healthcare facilities. While digital transformation introduces new requirements for both patients and providers, the efficiency benefits appear to outweigh these considerations. The observed variations in processing time patterns between traditional and digital systems highlight the fundamental differences in their operational mechanisms.

Infrastructure considerations remain paramount for successful implementation, as digital systems rely on technological foundations that may require strengthening in certain contexts. These findings highlight the role of digital health interventions in enhancing healthcare service delivery. However, their success depends on reliable internet infrastructure, staff training, and patient digital literacy. This healthcare modernization initiative exemplifies the broader potential of digital transformation in public service delivery. The insights gained from this assessment could inform strategic planning for healthcare administrators and policymakers looking to enhance operational efficiency. Future explorations should examine sustainability aspects, geographical variations, and demographic factors influencing system adoption to further optimize healthcare accessibility across diverse settings.

## Appendices

**Table 3:** Data collection template for time series analysis of conventional offline OPD registration process. OPD: outpatient department.

Cyclic elements (min)	Conventional offline OPD registration				
Elements (cycle count)	1/Entry	2/Waiting line	3/Registration	Total time	Average cycle time
1					
2					
3					

**Table 4:** Data collection template for time series analysis of online ABHA OPD registration process. ABHA: Ayushman Bharat Health Account; OPD: outpatient department.

Cyclic elements (min)	Online ABHA OPD registration					
Elements (cycle count)	1/Entry	2/App download	3/Waiting line	4/Registration	Total time	Average cycle time
1						
2						
3						
